# Value of the Neutrophil-Lymphocyte Ratio in Predicting COVID-19 Severity: A Meta-analysis

**DOI:** 10.1155/2021/2571912

**Published:** 2021-10-08

**Authors:** Ying Wang, Jingyi Zhao, Lan Yang, Junhui Hu, Yinhui Yao

**Affiliations:** ^1^Department of Pharmacy, The Affiliated Hospital of Chengde Medical College, Chengde 067000, China; ^2^Department of Functional Center, Chengde Medical College, Chengde 067000, China; ^3^Institute of Chinese Materia Medica, Chengde Medical College, Chengde 067000, China

## Abstract

**Background:**

Coronavirus disease 2019 (COVID-19) is highly contagious and continues to spread rapidly. However, there are no simple and timely laboratory techniques to determine the severity of COVID-19. In this meta-analysis, we assessed the potential of the neutrophil-lymphocyte ratio (NLR) as an indicator of severe versus nonsevere COVID-19 cases.

**Methods:**

A search for studies on the NLR in severe and nonsevere COVID-19 cases published from January 1, 2020, to July 1, 2021, was conducted on the PubMed, EMBASE, and Cochrane Library databases. The pooled sensitivity, specificity, positive likelihood ratio, negative likelihood ratio, diagnostic odds ratio (DOR), and area under the curve (AUC) analyses were done on Stata 14.0 and Meta-disc 1.4 to assess the performance of the NLR.

**Results:**

Thirty studies, including 5570 patients, were analyzed. Of these, 1603 and 3967 patients had severe and nonsevere COVID-19, respectively. The overall sensitivity and specificity were 0.82 (95% confidence interval (CI), 0.77-0.87) and 0.77 (95% CI, 0.70-0.83), respectively; positive and negative correlation ratios were 3.6 (95% CI, 2.7-4.7) and 0.23 (95% CI, 0.17-0.30), respectively; DOR was 16 (95% CI, 10-24), and the AUC was 0.87 (95% CI, 0.84-0.90).

**Conclusion:**

The NLR could accurately determine the severity of COVID-19 and can be used to identify patients with severe disease to guide clinical decision-making.

## 1. Introduction

Coronavirus disease 2019 (COVID-19) is an acute respiratory tract infection caused by the severe acute respiratory syndrome coronavirus 2 (SARS-CoV-2) [1]. The main routes of transmission of this highly contagious virus include respiratory droplets and close contact. The SARS-CoV-2 infection is associated with a wide range of clinical symptoms, including asymptomatic, nonsevere, and severe forms, which can rapidly lead to death [2]. Current evidence regarding COVID-19 pneumonia suggests that there may be an imbalance in the immune response that leads to high levels of inflammation in patients with severe pneumonia [3]. Therefore, laboratory parameters must be considered to diagnose COVID-19 and categorize patients as having nonsevere or critical disease, to plan the appropriate treatment and reduce mortality.

A study reported that patients with severe pneumonia had a lower lymphocyte count and a lower percentage of helper T cells, as well as slow lymphocyte recovery/lower number of lymphocytes during treatment. This may be closely related to virus-mediated immune paralysis [2]. The findings indicate that the progressive decrease in peripheral blood lymphocyte count could be an early clinical marker of severe COVID-19; however, dynamic observation of this experimental indicator is required. Moreover, the severity of the disease cannot be evaluated with this marker at an early stage, which is a limitation. In recent years, the neutrophil-lymphocyte ratio (NLR) has been considered one of the emerging markers of immune injury and inflammatory reaction [4, 5]. The sensitivity and specificity of this marker are better than that of white blood cell count (WBC), rise time is earlier, and its duration of persistence is longer [6]. In addition, the NLR can be used to assess the severity of infectious diseases and as an early warning marker of sepsis in critically ill patients [7]. Currently, there are many reports on the application of the NLR in the prediction of COVID-19 severity [8–37]. The purpose of this study was to investigate the potential of the NLR in the classification of disease severity in patients with COVID-19.

## 2. Methods

### 2.1. Literature Search

A literature search for articles published from January 1, 2020, to July 1, 2021, was performed on the PubMed, EMBASE, and Cochrane Library databases by two authors using the search terms “Neutrophil-to-lymphocyte ratio” or “NLR,” “Coronavirus disease 2019” or “novel Coronavirus disease 2019” or “SARS-CoV-2” or “Severe acute respiratory syndrome coronavirus 2.”

### 2.2. Inclusion and Exclusion Criteria

Data on the NLR was obtained from the examination records of patients admitted to hospitals in different countries to confirm the diagnosis of COVID-19. The inclusion criteria were as follows: (1) studies comprising patients diagnosed with COVID-19, with severe patients characterized by an oxygen saturation of 93% or less and/or <300 mmHg weighted oxygen pressure (PaO_2_)/fraction of inspired oxygen (FiO_2_); (2) studies with patients who could be divided into critical and noncritical groups based on the above criteria; and (3) studies from which the values of sensitivity and specificity could be obtained directly or indirectly via calculation. The exclusion criteria were studies that did not contain valid data, letters, case reports, and review articles. Two authors independently evaluated the selected literature based on the aforementioned inclusion and exclusion criteria, and any disagreements were resolved by discussion with a third author and consensus.

### 2.3. Data Extraction

The following data were extracted from the included literature independently by the two authors: first author, publication year, country of study, number of severe and nonsevere cases, sensitivity, specificity, study type, research center, age, and cut-off values. Differences in the extracted data between the two authors were resolved by the third author.

### 2.4. Quality Assessment

The quality assessment of diagnostic accuracy studies-2 (QUADAS-2) tool was used for quality assessment of the included literature to ensure the reliability and stability of the results of this meta-analysis [38].

### 2.5. Statistical Analysis

Stata 14.0 and Meta-disc 1.4 were used for statistical analyses [39, 40]. Sensitivity, specificity, positive likelihood ratio, negative likelihood ratio, diagnostic odds ratio (DOR), and area under the summary receiver operator characteristic curve (AUC) were calculated to assess the potential of the NLR in predicting the severity of COVID-19. Heterogeneity of the included studies was assessed using the *Q* or *I*^2^ test, where *p* < 0.05 or *I*^2^ ≥ 50% indicated potential heterogeneity. In the presence or absence of heterogeneity, the random effects or fixed effects models were adopted, respectively. Sensitivity analysis was performed to assess the robustness of the study. This meta-analysis was limited to the literature with a low risk of bias by excluding identified sources of heterogeneity. Deeks' funnel plot was used to evaluate the publication bias of the studies.

## 3. Results

### 3.1. Data Selection and Study Characteristics

**Figure 1:**
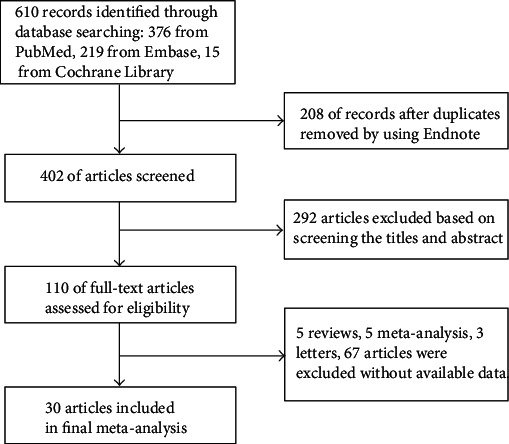
Study selection flow chart.

The literature search returned 610 articles, of which 208 were excluded due to duplication. After reading the titles and abstracts of the remaining studies, 292 were excluded. Upon full-text review of the remaining 110 studies, 80 were excluded due to insufficient relevant data, leaving 30 studies that met our inclusion criteria [8–37]. This process is shown in Figure 1.

There were a total of 5570 patients in the included studies (summarized in Table 1). Of these, 1603 and 3967 were severe and nonsevere cases, respectively. The diagnosis of COVID-19 was confirmed in all cases. The studies were conducted in China [8–13, 15, 16, 18, 19, 21–23, 25, 31–34], Pakistan [28], Argentina [20], Turkey [14, 17, 33, 35], Saudi Arabia [24], Iran [27, 36], and Egypt [29, 30]. The studies were conducted at a single center, with the exception of two multicenter studies [12, 13, 24, 34]. Two studies were prospective [11, 26], two were cross-sectional [28, 29], and the rest were retrospective studies.

### 3.2. Quality Assessment

**Figure 2:**
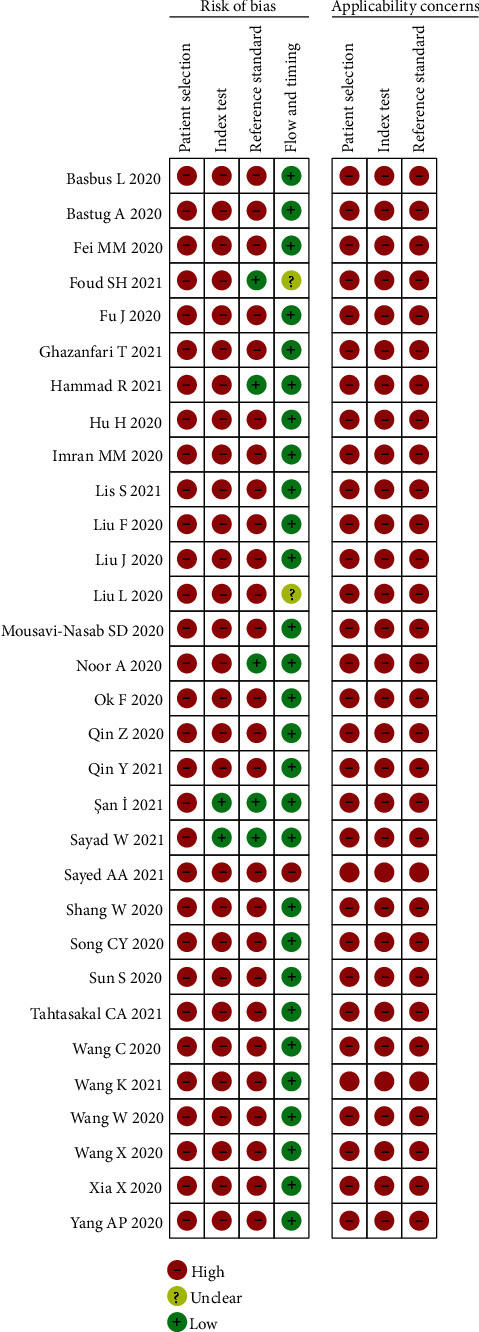
Risk of bias and applicability concerns in the included studies.

Quality assessment of all included studies was done using the QUADAS-2 tool (Figure 2).

### 3.3. Accuracy of the NLR in Diagnosing Severe COVID-19

**Figure 3:**
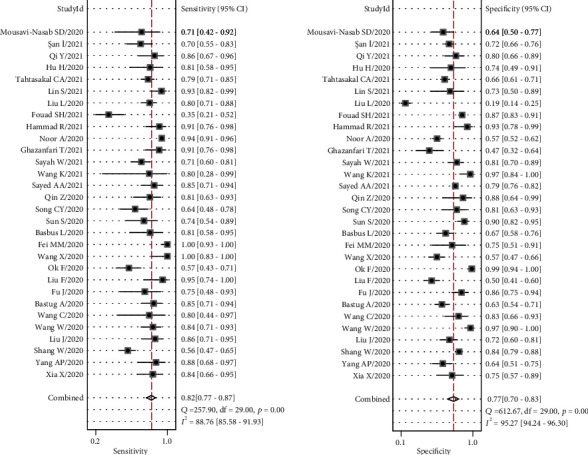
Forest plots for the sensitivity and specificity of the NLR in predicting COVID-19 severity.

**Figure 4:**
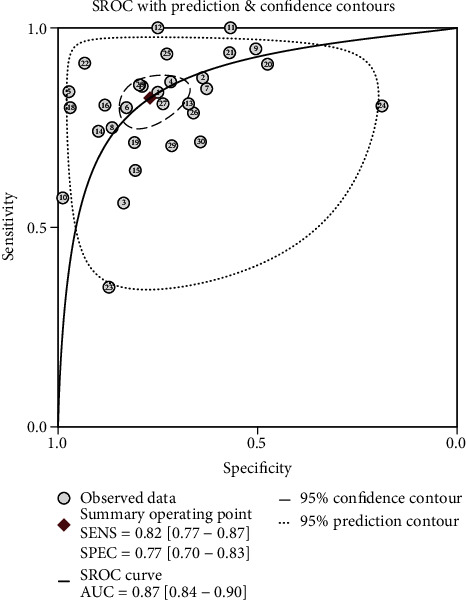
Symmetrical summary receiver operator characteristic curve of the NLR in all 30 studies.

Heterogeneity analysis revealed that the *I*^2^ values of sensitivity and specificity were 88.76 (95% confidence interval (CI), 85.58-91.93) and 95.27 (95% CI, 94.24-96.30), respectively, and both *p* values were <0.001, indicating significant interstudy heterogeneity. The overall sensitivity and specificity of the NLR in predicting severe COVID-19 cases were 0.82 (95% CI, 0.77-0.87) and 0.77 (95% CI, 0.70-0.83), respectively. The positive likelihood ratio, negative likelihood ratio, and DOR were 3.6 (95% CI, 2.7-4.7), 0.23 (95% CI, 0.17-0.30), and 16 (95% CI, 10-24), respectively. The AUC was 0.87 (95% CI, 0.84-0.90) implying that the NLR could accurately predict severe COVID-19 cases (Figures 3 and 4).

**Figure 5:**
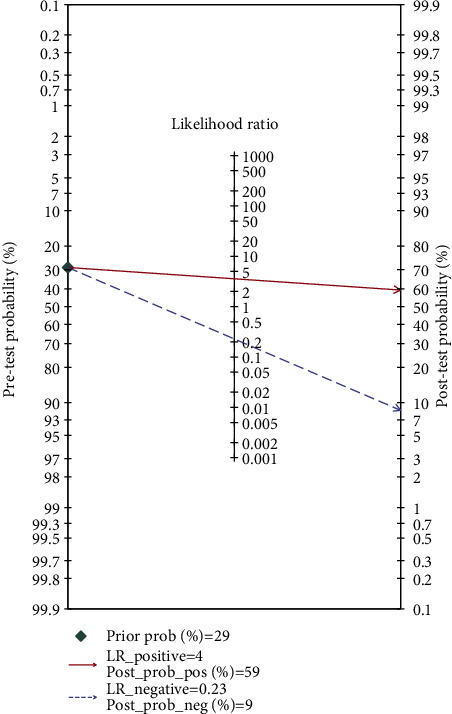
Fagan nomogram of the NLR for the prediction of COVID-19 severity.

Fagan's nomogram analysis of the pretest probability of severe COVID-19 by NLR changing revealed a posttest probability of 0.29 (Figure 5). When the positive of NLR was 4, the posttest positive probability in severe COVID-19 cases increased to 0.59, while the posttest probability of relative negative results fell to 0.09.

### 3.4. Subgroup and Metaregression Analyses

**Figure 6:**
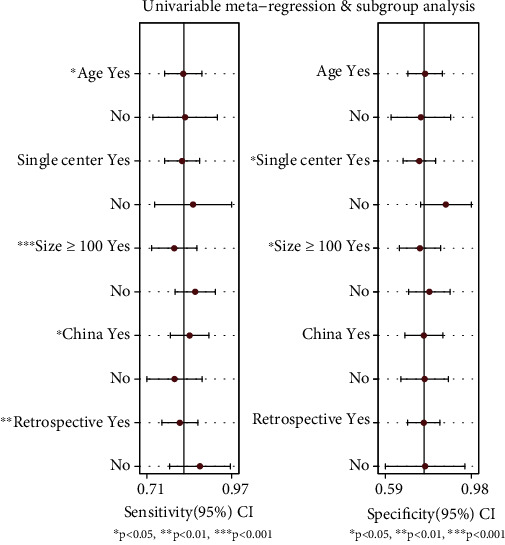
Univariable metaregression and subgroup analyses.

We performed a metaregression analysis to identify the sources of significant heterogeneity in the studies. Country-based differences (China or non-China), sample size (≥100 or <100), study type (retrospective or not), research center (single-center or multicenter), and age group differences (yes or no) were identified as potential sources of differences between trial designs or patients (Figure 6). Sample size, country-based differences, study type, and age differences were the main sources of heterogeneity in the sensitivity, while research center and sample size were the main sources of heterogeneity in the specificity. Additionally, diagnostic threshold analysis revealed a *p* value of <0.05, indicating the threshold effect as a potential source of heterogeneity.

### 3.5. Robustness Analysis and Publication Bias

**Figure 7:**
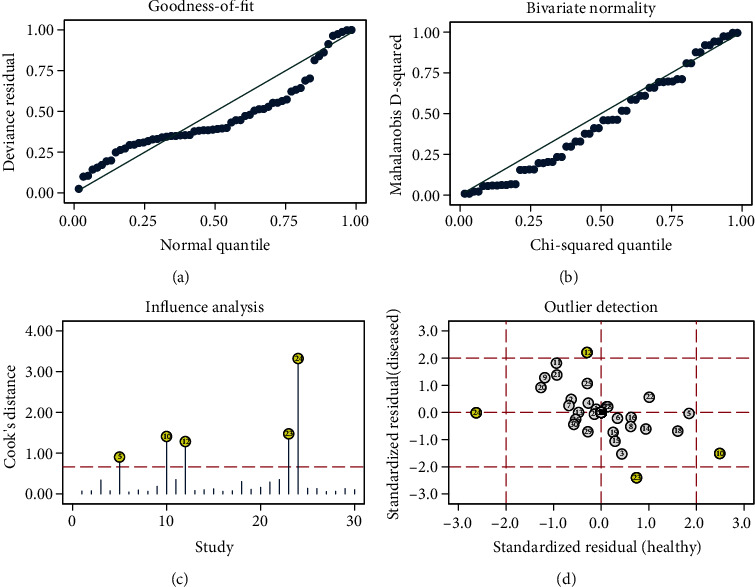
Stability and robustness analysis of the included studies: (a) goodness-of-fit; (b) bivariate normality; (c) influence analyses; (d) outlier detection.

Sensitivity analyses were performed to evaluate the reliability of the study results (Figure 7). The validity and robustness of the statistical analysis models were verified through goodness-of-fit, bivariate normality, and influence analyses, as well as the outlier detection method. The results obtained by eliminating these anomalies [12, 17, 19, 30, 31] did not differ significantly compared to the previous outcomes (AUC = 0.85).

**Figure 8:**
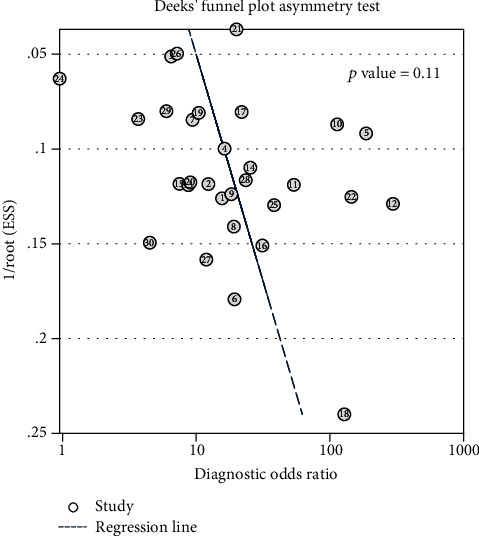
Deeks' funnel plot.

Deeks' funnel plot analysis of the 30 studies did not reveal any publication bias (*p* = 0.11, Figure 8).

## 4. Discussion

COVID-19, an infectious disease caused by SARS-CoV-2, mainly targets the lungs and in severe cases may result in multiorgan injury and death. SARS-CoV-2 binds to the alveolar ACE2 receptors and induces the release of inflammatory factors, which in turn activate the immune system, leading to a cytokine storm [41, 42]. Thus, timely and accurate identification of severe COVID-19 cases after diagnosis is important for the immediately treatment of high-risk patients. Significantly lower lymphocyte and higher neutrophil counts have been observed in patients with severe COVID-19 compared to those with mild disease [2]. A study suggested that the NLR could effectively distinguish between severe and nonsevere COVID-19 cases [43]. In this study, we evaluated the accuracy of the NLR in predicting the severity of COVID-19.

The NLR is a simple, economical, commonly used, and rapidly available hematological assay. Lagunas-Rangel conducted a meta-analysis and reported that the NLR, as an indicator of inflammation, predicted the severity of COVID-19; however, the sample size of the study was small [44]. In this study, we conducted a meta-analysis of 30 studies to evaluate the role of the NLR in predicting the severity of COVID-19 at admission. The results of our analysis revealed that the sensitivity, specificity, and AUC values of the NLR were 0.82, 0.77, and 0.87, respectively, indicating that the ratio could distinguish severe COVID-19 cases from mild cases with high accuracy. The positive and negative likelihood ratios were 3.6 and 0.23, respectively, indicating that the NLR had a moderate capacity to identify critically ill and noncritically ill patients. A DOR value of 16 demonstrated the high capacity of the NLR to accurately identify severe COVID-19 cases. Taken together, these data indicate that the NLR has a high capacity to accurately predict the severity of COVID-19.

The QUADAS-2 tool revealed that the risk of bias in the majority of the studies was high. Of the 30 studies included, two each were cross-sectional and prospective studies, while the remaining 26 were retrospective. In a retrospective study design, the accuracy of a diagnostic test may be overestimated because the patients are considered unrepresentative [45]. The index test results are explained in the reference results that are already known. In terms of applicability, based on the results of each study and this meta-analysis, the performance of the NLR was found to be favorable in predicting the severity of COVID-19.

We performed a metaregression analysis to explore the sources of potential heterogeneity in the studies. The analysis revealed that the difference in age between patients with severe and nonsevere disease was the main source of heterogeneity in the sensitivity. It is well known that physiological and immune functions gradually decline in the elderly [46]. Therefore, elderly patients with COVID-19 are more likely to develop severe disease [47, 48]. A study reported that the volume of the thymus in individuals over the age of 60 years was only one-tenth of that in young individuals, with negligible naïve T cells for maintenance of the peripheral immune system [49]. Therefore, the resistance to new viral infections is weak in the elderly. The expression of ACE2 in different tissues and joint analysis of the immune characteristics in the elderly revealed that different hosts demonstrated variable immune responses, increased disease severity, and higher mortality. The differences in the ACE2 gene are not only related to the age of the patients but also the race [50, 51]. This is one of the reasons why the inclusion of regional differences in the meta-analysis was a source of heterogeneity. In addition, the sample size and research center (single-center or multicenter) were a major source of heterogeneity in the sensitivity and specificity, respectively. Studies have shown that when the effect size is the average difference, sampling error does not cause significant deviation; however, it affects the standardized average difference and odds ratio. The overall odds ratio and risk ratio demonstrate significant deviation even if the sample size exceeds 50, which may lead to bias in the results of the meta-analysis [52]. The threshold analysis shows that the threshold of this study is one of the reasons for the heterogeneity of meta-analysis. Among the 30 studies included in this meta-analysis, the cut-off value of the NLR for the severity classification of COVID-19 patients ranged from 1 to 13.39. The influencing factors of the NLR are closely related to the physical condition of the patients. The number of elderly patients with severe COVID-19 infection was very high. Moreover, these patients demonstrated more complications that were related to the values of the NLR. For example, the number of patients with rheumatoid arthritis was higher than those without any disease, and the NLR was related to the progression of the rheumatoid arthritis [12]. Although the NLR of newly admitted patients was considered, the nutritional status of a patient varies according to the standard of living in different regions. Some studies have reported that malnutrition is an established risk factor for COVID-19 that demonstrates the strongest relationship with the NLR [53, 54]. Considering the lack of unification in the NLR threshold, the value to determine the severity of COVID-19 needs to be considered depending on the clinical situation. Our assessment did not reveal any publication bias in this meta-analysis. Additionally, sensitivity analysis did not reveal any significant changes upon exclusion of the outlier results.

Our findings show that the NLR has a high capacity to accurately predict the severity of COVID-19. However, this study has certain limitations. First, the majority of the included studies are from China, which limits the generalizability of the results, since the virus may have different effects based on genetic and environmental factors [55]. Second, further clinical studies are necessary considering the heterogeneity in the included studies.

## 5. Conclusions

In conclusion, our meta-analysis revealed that the NLR has a high capacity to accurately predict the severity of COVID-19, which can permit laboratory-based differentiation of nonsevere and severe cases. However, further studies are needed to confirm these findings by including patients from different ethnic backgrounds and geographic regions.

## Figures and Tables

**Table 1:** Characteristics of the studies included in the meta-analysis. Note: true positive (TP), true negative (TN), false positive (FP), and false negative (FN).

First author	Year	Country	Severe	Nonsevere	Sensitivity	Specificity	TP	FP	FN	TN	Median age (severe/nonsevere)	Centers	Optimal cut-off	Study type
Xia X [8]	2020	China	31	32	0.84	0.75	26	8	5	24	64.55/62.25	Single	4.795	Retrospective
Yang AP [9]	2020	China	24	69	0.88	0.63	21	25	3	44	57.9/42.1	Single	3.3	Retrospective
Shang W [10]	2020	China	139	304	0.56	0.84	78	50	61	254	64/58	Single	4.283	Retrospective
Liu J [11]	2020	China	37	78	0.87	0.72	32	22	5	56	NA	Single	3.13	Prospective
Wang W [12]	2020	China	50	73	0.84	0.97	42	2	8	71	79.5/61.0	Multicenter	4.16	Retrospective
Wang C [13]	2020	China	10	35	0.83	0.82	8	6	2	29	43/38	Multicenter	13.39	Retrospective
Bastug A [14]	2020	Turkey	46	145	0.84	0.62	39	54	7	91	71/43	Single	3.21	Retrospective
Fu J [15]	2020	China	16	59	0.75	0.86	12	8	4	51	51.8/45.1	Single	—	Retrospective
Liu F [16]	2020	China	19	115	0.94	0.50	18	57	1	58	63/50	Single	—	Retrospective
Ok F [17]	2020	Turkey	54	85	0.57	0.98	31	1	23	84	68.3/47.2	Single	5.72	Retrospective
Wang X [18]	2020	China	20	111	1	0.56	20	48	0	63	NA	Single	2.306	Retrospective
Fei MM [19]	2020	China	52	20	1	0.73	52	5	0	15	64/55.7	Single	3	Retrospective
Basbus L [20]	2020	Argentina	21	110	0.81	0.67	17	36	4	74	77/47	Single	3	Retrospective
Sun S [21]	2020	China	27	89	0.74	0.89	20	9	7	80	62/47	Single	4.5	Retrospective
Song CY [22]	2020	China	42	31	0.64	0.81	27	6	15	25	55.5/34	Single	5.87	Retrospective
Qin Z [23]	2020	China	31	17	0.81	0.88	25	2	6	15	61/45	Single	3.55	Retrospective
Sayed AA [24]	2021	Saudi Arabia	41	660	0.86	0.79	35	139	6	521	45/35	Multicenter	5.5	Retrospective
Wang K [25]	2021	China	5	33	0.85	0.98	4	1	1	32	60/45	Single	4.425	Retrospective
Sayah W [26]	2021	Algeria	80	73	0.72	0.8	57	14	23	59	65/57	Single	5.9	Prospective
Ghazanfari T [27]	2021	Iran	33	40	0.9	0.47	30	21	3	19	59.14/52.78	Single	3.53	Retrospective
Noor A [28]	2020	Pakistan	370	365	0.94	0.57	347	157	23	208	48.11/44.47	Single	—	Cross-sectional
Hammad R [29]	2021	Egypt	34	30	0.91	0.93	31	2	3	28	60/27	Single	3.1	Cross-sectional
Fouad SH [30]	2021	Egypt	40	298	0.34	0.87	14	38	26	260	46.8	Single	7.53	Retrospective
Liu L [31]	2020	China	92	202	0.8	0.19	74	164	18	38	62/50.1	Single	5	Retrospective
Lin S [32]	2021	China	46	22	0.93	0.73	43	6	3	16	56.4/44	Single	3.63	Retrospective
Tahtasakal CA [33]	2021	Turkey	136	398	0.79	0.66	107	135	29	263	66/59	Single	3.69	Retrospective
Hu H [34]	2020	China	21	19	0.81	0.74	17	5	4	14	63/43	Single	3.84	Retrospective
Qi Y [35]	2021	China	28	54	0.86	0.79	24	11	4	43	55.5/40	Multicenter	3.531	Retrospective
Şan İ [36]	2021	Turkey	44	344	0.71	0.71	31	98	13	246	67.5/42	Single	3.59	Retrospective
Mousavi-Nasab SD [37]	2020	Iran	14	56	0.71	0.64	10	20	4	36	41.3/43	Single	1	Retrospective

## Data Availability

The data of Table 1 used to support the findings of this study are included within the article (see References).
